# An Updated Systematic Review and Meta-analysis of the Impact of Graduated Compression Stockings in Addition to Pharmacological Thromboprophylaxis for Prevention of Venous Thromboembolism in Surgical Inpatients

**DOI:** 10.1097/SLA.0000000000006096

**Published:** 2023-09-27

**Authors:** Benedict R.H. Turner, Matthew Machin, Marwah Salih, Sara Jasionowska, Rebecca Lawton, Francesca Siracusa, Adam M. Gwozdz, Joseph Shalhoub, Alun H. Davies

**Affiliations:** Department of Surgery and Cancer, Section of Vascular Surgery, Imperial College London, London, UK

**Keywords:** deep vein thrombosis, graduated compression stockings, pharmacological prophylaxis, pulmonary embolism, thromboprophylaxis, venous thromboembolism

## Abstract

**Objective:**

To compare the rate of venous thromboembolism (VTE) in surgical inpatients with pharmacological thromboprophylaxis and additional graduated compression stockings (GCSs) versus pharmacological thromboprophylaxis alone.

**Background:**

Surgical inpatients have elevated VTE risk; recent studies cast doubt on whether GCS confers additional protection against VTE, compared with pharmacological thromboprophylaxis alone.

**Methods:**

The review followed “Preferred Reporting Items for Systematic Reviews and Meta-analyses” guidelines using a registered protocol (CRD42017062655). The MEDLINE and Embase databases were searched up to November 2022. Randomized trials reporting VTE rate after surgical procedures, utilizing pharmacological thromboprophylaxis, with or without GCS, were included. The rates of deep venous thrombosis (DVT), pulmonary embolism, and VTE-related mortality were pooled through fixed and random effects.

**Results:**

In a head-to-head meta-analysis, the risk of DVT for GCS and pharmacological thromboprophylaxis was 0.85 (95% CI: 0.54–1.36) versus for pharmacological thromboprophylaxis alone (2 studies, 70 events, 2653 participants). The risk of DVT in pooled trial arms for GCS and pharmacological thromboprophylaxis was 0.54 (95% CI: 0.23–1.25) versus pharmacological thromboprophylaxis alone (33 trial arms, 1228 events, 14,108 participants). The risk of pulmonary embolism for GCS and pharmacological prophylaxis versus pharmacological prophylaxis alone was 0.71 (95% CI: 0.0–30.0) (27 trial arms, 32 events, 11,472 participants). There were no between-group differences in VTE-related mortality (27 trial arms, 3 events, 12,982 participants).

**Conclusions:**

Evidence from head-to-head meta-analysis and pooled trial arms demonstrates no additional benefit for GCS in preventing VTE and VTE-related mortality. GCS confer a risk of skin complications and an economic burden; current evidence does not support their use for surgical inpatients.

Venous thromboembolism (VTE) is a common and serious complication of hospitalization, carrying a high morbidity and mortality. European epidemiological studies report an overall incidence of 110 to 130 cases of VTE per 10,000 patients per year,^1^ with 10% to 12% of deaths per annum being VTE-related.^2,3^ Of the observed VTE cases, 50% to 60% are associated with hospitalization and there is a >100-fold increase in the incidence of VTE in hospital inpatients versus community residents.^1^ For surgical inpatients, postoperative immobilization and hypercoagulable states due to inflammation confer a 128% elevated VTE risk compared with medical inpatients.^3,4^ The ENDORSE study demonstrated that the proportion of surgical inpatients at high risk of VTE by American College of Chest Physicians criteria was 64.4% compared with 41.5% of medical inpatients.^5^ Furthermore, recurrence of deep venous thrombosis (DVT) and pulmonary embolism (PE) is common (18.5% and 4.4% cumulative risk over 5 years, respectively)^2^; and long-term complications, such as postthrombotic syndrome and pulmonary hypertension, are difficult to manage, with a significant impact on quality of life.^2^ As a result of the high incidence, symptom morbidity, and disease chronicity, the cost of managing VTE in the USA is estimated at 7 to 10 billion US dollars per annum.^6^

Thromboprophylaxis for hospital inpatients can be divided into pharmacological measures: anticoagulation with low molecular weight heparin (LMWH), and mechanical measures: graduated compression stockings (GCSs) or intermittent pneumatic compression devices (IPCD). Mechanical VTE prophylaxis is thought to function by increasing the volume and velocity of blood flow in the lower limbs to reduce venous stasis.^1^ For most surgical inpatient groups, the “National Institute for Health and Care Excellence” guidelines recommend a combination of mechanical and pharmacologic prophylaxis.^7^ Selection between GCS and IPCD is left to clinical judgment in the context of orthopedic, major abdominal, and thoracic operations.^8^ The “American College of Chest Physicians” and “American Society of Hematology” guidelines recommend dual mechanical and pharmacological prophylaxis for high-risk general surgical inpatients but do not distinguish between pharmacological thromboprophylaxis measures in numerous circumstances.^9^

Over the last decade, several studies have aimed to elucidate whether GCS has a useful role in thromboprophylaxis. In 2009, the CLOTS 1 randomized control trial was one of the first to demonstrate, in a cohort of stroke patients, that GCS in addition to standard of care conferred no significant reduction in risk of DVT when compared with standard of care alone.^10^ Subsequently, in 2015, a systematic review by Mandavia et al^11^ concluded from the available literature that there was no clear additional benefit of GCS to pharmacological VTE prophylaxis for surgical inpatients. Since 2015, the body of evidence has continued to grow, thus the aim of this article was to undertake a systematic review and meta-analysis of the benefit of pharmacological prophylaxis and GCS versus pharmacological prophylaxis alone for the prevention of VTE in surgical inpatients. This remains a highly relevant issue in the context of financially unstable postpandemic health care systems; abandoning the use of GCS would represent a significant change in current practice.

## METHODS

This systematic review was performed as an update to a published peer-reviewed article, followed the same protocol (PROSPERO: CRD42017062655),^11^ and adhered to “Preferred Reporting Items for Systematic Reviews and Meta-analyses” guidelines.

### Search Strategy

Online searches of the MEDLINE and EMBASE databases through the Ovid platform were performed up to November 2022. There were no restrictions imposed on the search regarding publication type or language at the search stage. Search terms were as published^11^ and compromised of keywords for VTE and encompassed generic and branded drug names of pharmacologic prophylaxis. References and appendixes of all included articles were also screened to further identify any eligible articles. Articles identified by the search were independently screened using the title and abstract against the inclusion criteria by 2 reviewers (B.R.H.T. and S.J.). Any discrepancies were discussed between the reviewers. If the discrepancy failed to be resolved, it was discussed with the lead author (A.H.D.).

### Eligibility Criteria

Articles reported on randomized controlled trials undertaken in secondary care, that is, surgical inpatients. Patients were admitted to the hospital for either elective or emergency surgery and were randomized to receive either pharmacologic prophylaxis alone or pharmacologic prophylaxis in addition to GCS until discharge. Full inclusion and exclusion criteria are published in Mandavia et al.^11^

### Data Extraction and Analysis

Data from the included articles was extracted using a standardized template in Microsoft Excel by 2 reviewers (B.R.H.T. and S.J.) independently. Participant, thromboprophylaxis, and diagnostic investigation data were collected, as well as the rate of DVT, PE, and VTE-related mortality and complications. Statistical analyses were computed using R version 3.3.2 (R Core Team, GNU GPL v2 License), R Studio version 1.0.44 (RStudio Inc., GNU Affero General Public License v3), and RevMan5 (The Cochrane Collaboration). Where data from 2-armed trials were available, they were pooled with fixed effects through a head-to-head meta-analysis. Where data were collected from trial arms, they were pooled using a random-effect model and meta-proportions analysis. Where overall proportions were low, a logit transformation was used to normalize the data, and where there were high proportions of zero event rates, the double arcsine-Tukey transformation was performed.^12^ Heterogeneity was computed with the *I* ^2^ statistic with further subgroup analysis planned in the presence of significant statistical heterogeneity.

### Risk of Bias and Quality Assessment

The risk of bias was assessed using the Cochrane Risk of Bias tool. This was performed independently by 2 reviewers (B.R.H.T. and A.M.G.). Any discrepancies were mediated by a third reviewer (M.M.). The “Grading of Recommendations, Assessment, Development, and Evaluations” framework was used to assess the quality and certainty of the evidence, using the GradePro (Evidence Prime Inc.) online platform.

## RESULTS

**FIGURE. 1:**
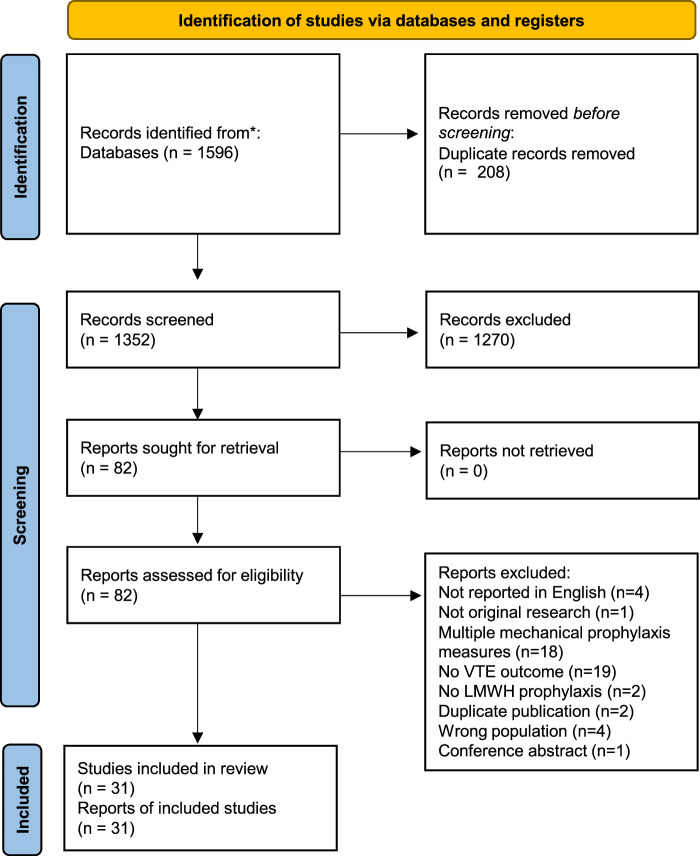
PRISMA flow diagram of studies included in the systematic review and meta-analysis. PRISMA indicates preferred reporting items for systematic reviews and meta-analyses.

Between January 2005 and November 2022, there were 1570 articles that matched the search criteria, with 204 duplicates removed. Of 1366 articles screened, 76 proceeded to full-text review after which 31 articles were included in the final text (Fig. 1). Supplemental Table (Supplemental Digital Content Table 1, http://links.lww.com/SLA/E865) demonstrates the full summary of the included study characteristics. Baseline characteristics, such as age and sex, were balanced in the studies. There were 14,108 total participants, 2205 of whom received GCS and pharmacological prophylaxis, whereas 11,903 received pharmacological prophylaxis alone. A range of pharmacological thromboprophylaxis regimes were implemented and can be observed alongside the study characteristics in the Supplemental Table (Supplemental Digital Content Table 1, http://links.lww.com/SLA/E865). The most frequent regime observed was enoxaparin 40 mg once daily and pharmacological prophylaxis was administered for a minimum of 2 days and a maximum of 2 months, though always until discharge.

With regard to surgical characteristics, there were 22 orthopedic trial arms,^13–33^ 3 gynecologic/pelvic,^34–36^ 4 with major abdominal surgery,^37–40^ and 2 studies with mixed elective surgical populations.^18,41^ Six trial arms considered both emergency and elective surgical patients in their recruitment, 3 considered only emergency patients, and 22 trials/trial arms considered solely elective cohorts. DVT was diagnosed symptomatically in the majority of studies but always confirmed with ultrasound or venography. PE was diagnosed using computed tomography pulmonary angiography, spiral computed tomography, pulmonary scintigraphy, and ventilation-perfusion scanning.

### Deep Venous Thrombosis

**FIGURE. 2:**
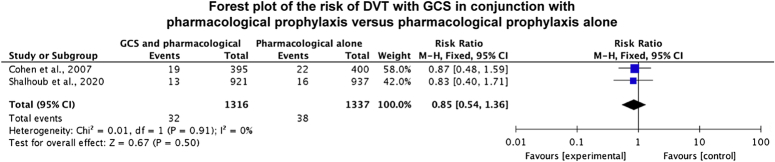
Forest plot of the risk of DVT with GCS in conjunction with pharmacological prophylaxis versus pharmacological prophylaxis alone.

Two studies directly compared GCS and pharmacological prophylaxis with pharmacological prophylaxis alone.^5,41^ For the outcome of DVT, there were 32 events in 1316 participants for the GCS and pharmacological prophylaxis group versus 38 events in 1337 participants for the pharmacological prophylaxis alone group. The risk of DVT for GCS and pharmacological prophylaxis versus pharmacological prophylaxis alone was 0.85 (95% CI: 0.54–1.36; 2 studies, 70 events, 2653 participants; Fig. 2). Heterogeneity as reported through *I* ^2^ was 0%.

**FIGURE. 3:**
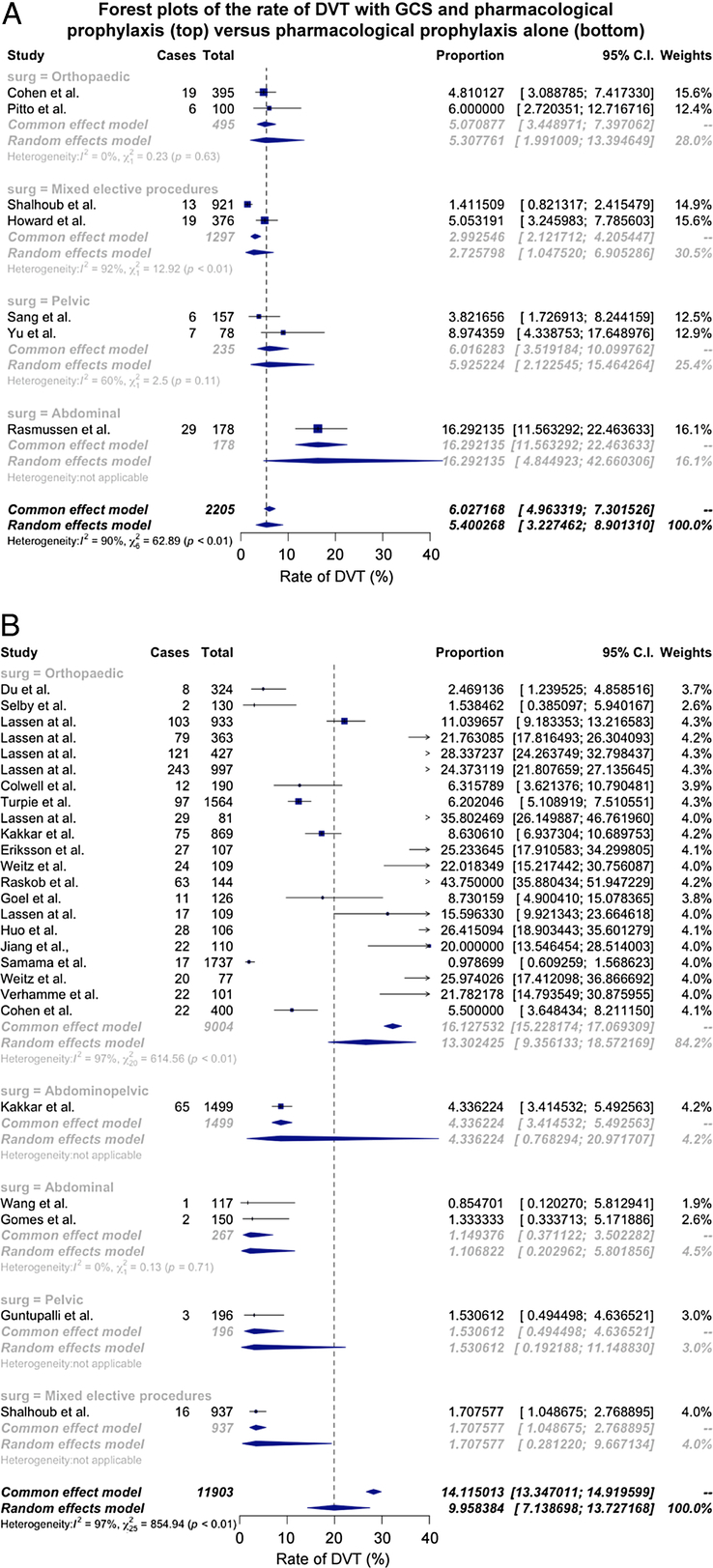
A, The pooled rate of DVT in studies using GCS in conjunction with pharmacological prophylaxis. B, The pooled rate of DVT in studies using pharmacological prophylaxis alone.

The rate of DVT was reported in 33 trial arms; for the GCS plus pharmacological prophylaxis group the pooled rate of DVT was 5.4% (95% CI: 3.2%–8.9%; 7 trial arms, 1129 events, 11,903 participants)^13,18,26,35,36,39,41^ versus 10.0% (95% CI: 7.1%–13.7%; 26 trial arms, 99 events, 2205 participants, *I* ^2^ = 90%)^13-17,19-25,27-33^ for pharmacological prophylaxis alone; the relative risk was 0.54 (95% CI: 0.23–1.25; Fig. 3). Significant statistical heterogeneity was noted. Exploratory analysis revealed that type of surgery significantly moderated the rate of DVT in the pharmacological prophylaxis alone group (*P* = 0.001) but not the pharmacological prophylaxis and GCS group (*P* = 0.15).

### Pulmonary Embolism

**FIGURE. 4:**
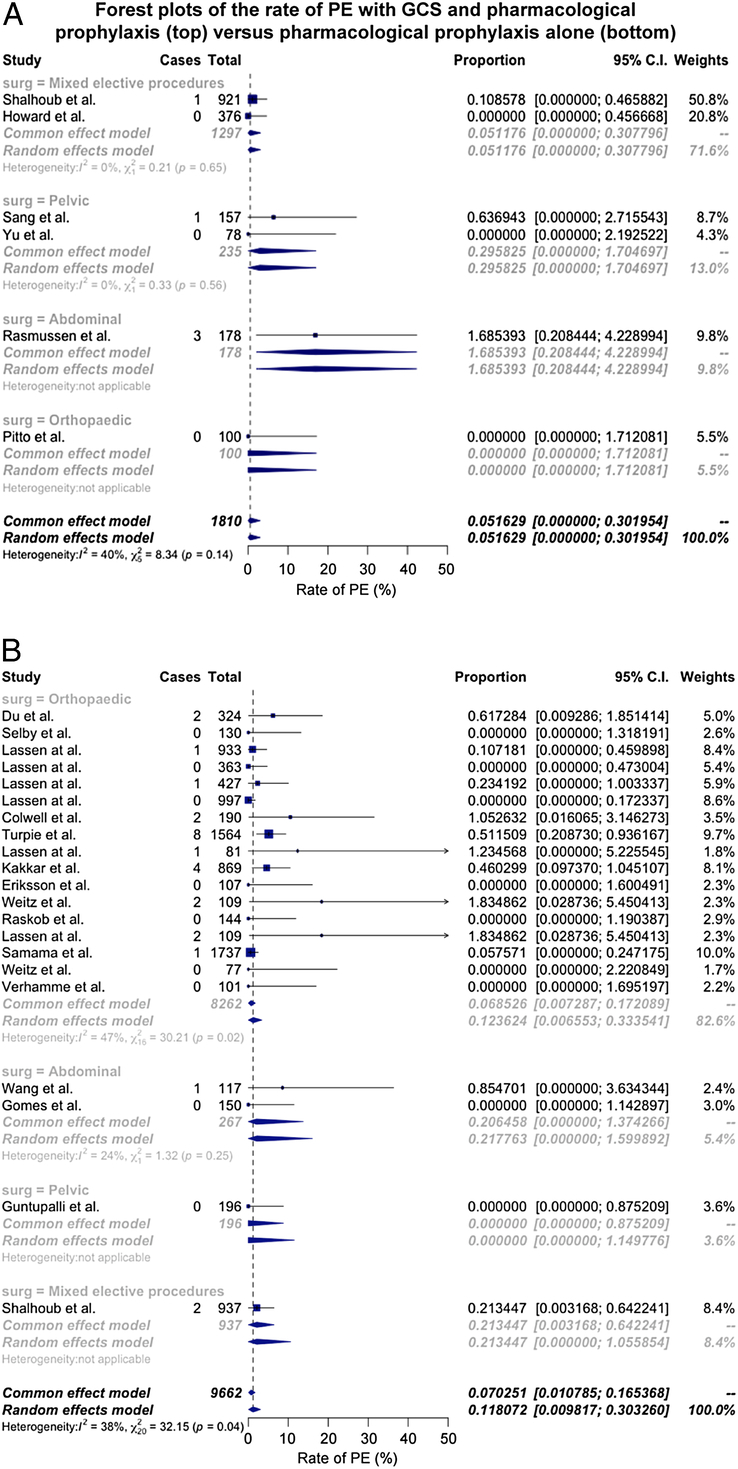
A, the pooled rate of PE in studies using GCS in conjunction with pharmacological prophylaxis. B, The pooled rate of PE in studies using pharmacological prophylaxis alone.

There were 2 studies, which compared the rate of PE for GCS and pharmacological prophylaxis with pharmacological prophylaxis alone.^5,41^ However, as one of the studies had an event rate of zero,^5^ conventional head-to-head meta-analysis was precluded. In pooled trial arms, the rate of PE with pharmacological prophylaxis and GCS group was 0.05% (95% CI: 0.00%–0.30%; 6 trial arms, 5 events, 1810 participants, *I* ^2^ = 40%)^18,26,35,36,39,41^ versus 0.11 (95% CI: 0.01%–0.30%; 21 trial arms, 27 events, 9662 participants, *I* ^2^ = 38%)^14–16,21–25,27–34,37,40,41^ in the pharmacological prophylaxis alone group (Fig. 4); the relative risk of PE was 0.71 (95% CI: 0.00–30.0).

### Venous Thromboembolism–Related Mortality

**FIGURE 5:**
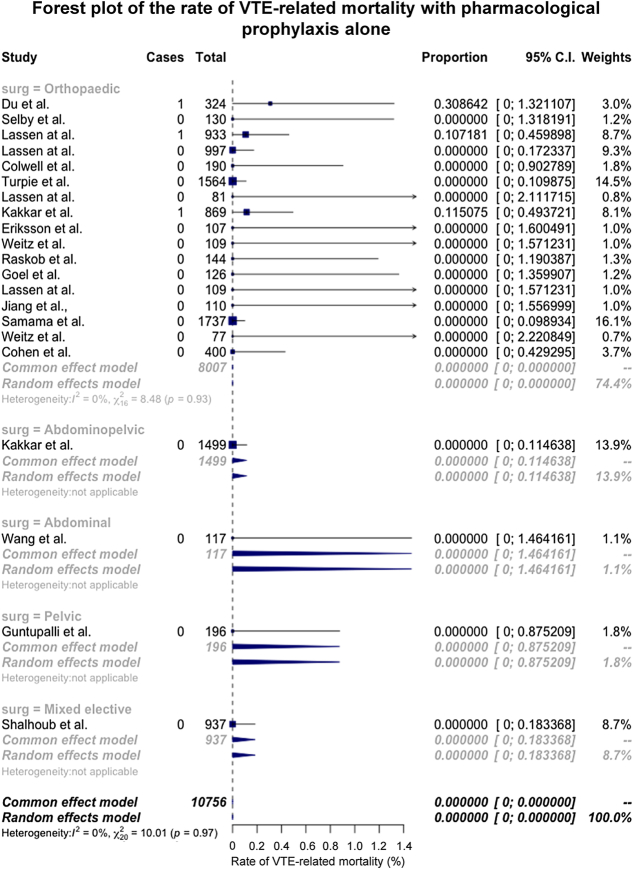
The pooled rate of VTE-related mortality in studies using pharmacological prophylaxis alone.

There were 27 trial arms that reported on VTE-related mortality. There were no deaths in the GCS and pharmacological prophylaxis group (6 trial arms, 0 events, 2226 participants, *I* ^2^ = 0%).^5,18,35,36,39,41^ In the pharmacological prophylaxis alone trial arms, the crude rate of VTE-related mortality was 0.03%; when pooled with meta-analysis, this fell to 0.00% (95% CI: 0.00%–0.00%; 21 trial arms, 3 events, 10,756 participants, *I* ^2^ = 0%)^13–16,21–25,27–30,32–34,40,41^ (Fig. 5).

### Adverse Events

Four trials reported adverse events related to stocking use.^13,18,35,41^ There were 67 minor adverse events across the trials that reported this outcome. In the first study, there were 7 unspecified adverse events in 391 patients (1.8%).^13^ In the second study, there were 3 adverse events in 376 patients (0.8%), all of which were minor foot abrasions.^18^ In the third study, there were no adverse events (0%) in 157 patients,^35^ and in the fourth study, there were 75 adverse events in 55 of 947 participants (5.8%) who received GCS.^41^

### Risk of Bias

Included studies were at a mix of low, unclear, and high risk of bias. Older studies tended not to have published protocols and were at unclear or high risk of selective reporting. Many of the trials demonstrated incomplete outcome data, in part, this was due to difficulties in ensuring all patients had venography in the applicable trials. This was accounted for in the risk of bias assessment, but differential attrition between individual trial arms or lack of adequate explanation for attrition were considered as introducing a high risk of bias. Blinding was performed well across the trials, including the use of a double-dummy approach in many trials. Even when blinding was considered not possible to implement, many trials opted for a single-blinded approach, and knowledge of the intervention by trial participants was considered to have a low risk of influencing the outcome of objectively diagnosed VTE. Randomization sequence generation was well performed but details regarding allocation concealment were often omitted. A full risk of bias assessment is available in the Supplemental Table (Supplemental Digital Content Table 2, http://links.lww.com/SLA/E865).

### Grading of Recommendations, Assessment, Development, and Evaluations Assessment

The quality of evidence from the 2 trials included in the head-to-head meta-analysis was graded as high-quality evidence, with good consistency, directness, precision, and low risk of bias. The quality of evidence for the pooled rate of PE and VTE-related mortality was downgraded to moderate quality evidence in light of the risk of bias. The quality of evidence for the pooled rate of DVT in trial arms was downgraded to very low in light of the serious risk of bias, imprecision, and inconsistency.

## DISCUSSION

This review is an update to the 2015 review into the additional benefit of GCS to pharmacological thromboprophylaxis in preventing VTE in surgical inpatients. It has considered a wealth of new high-quality evidence, including 1 head-to-head randomized controlled trial and 10 further randomized trial arms, with 4589 new participants included. Meta-analysis of only 2 head-to-head trials^13,41^ has generated high-quality evidence for the rates of DVT, PE, and VTE-related mortality with GCS and pharmacological prophylaxis versus pharmacological prophylaxis alone. Though there was some evidence that the DVT and PE rates were reduced with risk ratios of 0.85 and 0.54, respectively, the CIs demonstrated that the results were not statistically significant. This has been further supported by a meta-proportions analysis to pool VTE event rates and again demonstrate no difference between GCS and pharmacological prophylaxis alone versus pharmacological prophylaxis alone. Though the raw rate of VTE-related mortality was slightly higher (0.03%) in the pharmacological prophylaxis alone trial arms, there were nearly ten times the number of patients included with 15 more trials reporting this outcome and, after data pooling to account for participant numbers, there was no significant difference. Since the 2015 review, the GAPS trial has been published; a single-blind, pragmatic, randomized controlled trial of GCS with pharmacological prophylaxis (LMWH) versus pharmacological prophylaxis alone.^41^ It is the largest randomized trial performed to date and demonstrated the noninferiority of pharmacological prophylaxis alone (*P* < 0.001) as compared with prophylaxis with LMWH and GCS, in a representative cohort of UK elective surgical patients. Overall, the results of the meta-analysis support the hypothesis from the GAPS trial that GCS confer no additional benefit over standard pharmacological prophylaxis for surgical inpatients.

GCS was popularized for the prevention of DVT in the 1930s, before the advent of pharmacological prophylaxis. The present review considers evidence from randomized controlled trials in the recent era, which makes it more generalizable to the current population of surgical inpatients than older studies. Even so, a significant amount of clinical and statistical heterogeneity was observed between the studies. For example, in the pharmacological prophylaxis alone trial arms, there was a range of DVT rates from <1% to 43%^27,28^; both trials considered only elective orthopedic surgery. The former study by Samama and colleagues conducted a placebo-controlled, double-blind, randomized trial in which patients were anticoagulated with rivaroxaban or enoxaparin. Types of surgery included any elective orthopedic lower-limb surgery necessitating the use of thromboprophylaxis for more than 2 weeks. Patients received anticoagulation for at least 2 weeks, with a significant proportion of patients taking anticoagulation for >1 month, and in some cases, >2 months.^28^ Meanwhile, Raskob and colleagues conducted a double-blind, randomized trial in which patients undergoing elective total hip procedures and took dalteparin for 7 to 10 days with bilateral venography after this time to diagnose DVT. The VTE rate after total hip arthroplasty is reported as anything from 20% to 60%,^42,43^ whereas after knee ligament repair the rate of VTE may be as low as 0.4%.^44^ Therefore, differences between the intrinsic VTE risk of surgical procedures, as well as the duration of anticoagulation may account for some of the heterogeneity observed between studies. The proportions of trial arms considering elective orthopedic procedures were balanced between the arms of the meta-analysis and, therefore, all studies were included to generate the most representative overview of VTE rates possible. Clearly, the highest quality evidence is derived from a meta-analysis of the 2 head-to-head randomized controlled trials comparing GCS plus pharmacological prophylaxis with pharmacological prophylaxis alone.

The Commission for Quality and Innovation made tackling VTE, one of its key goals over the last decade, and initiatives have made VTE risk assessment and prescription of prophylaxis a routine part of all UK National Health Service (NHS) hospital pathways. As a result, by July 2013, 96% of adult hospital admissions in the NHS had been risk assessed for VTE compared with 50% in July 2010^7^. In the UK, it has been estimated that 70% of the inpatient population uses GCS.^13^ This represents a significant annual cost of buying and applying stockings, estimated at £63.1 million for surgical inpatients in England alone.^45^ Given these substantial costs, the financial pressure on the NHS, and the nonsignificant difference between GCS and pharmacological prophylaxis versus pharmacological prophylaxis alone, there is sufficient evidence to support removing the recommendation for GCS from guidelines in the context of elective surgery. In addition to having a financial cost, GCS is burdensome for patients; complication rates of up to 5% have been demonstrated in this review, including ulceration, rashes, and abrasions. This relatively high complication rate in part explains the generally low observed compliance with GCS,^46^ which further reduces their likely efficacy outside of the controlled clinical trial setting.

The strengths of this study stem from the meta-analysis of 2 head-to-head trials which were well designed with a low risk of bias and considered a total of 2600 participants across the studies. This has generated high-quality evidence that is further supported through the presentation of pooled rates of DVT, PE, and VTE-related mortality which show no difference between pharmacological prophylaxis and additional GCS versus pharmacological prophylaxis alone. Data on DVT, PE, and VTE-related mortality rates were pooled from randomized trial arms and hence the risk of confounding across trials should be reduced, however, included trial arms exhibited various degrees of bias, and the pooling of studies may have introduced a degree of clinical and statistical heterogeneity. Despite this, pooling trials has allowed us to generate an accurate rate of VTE which accounts for intrinsic differences in procedural VTE rate through grouping trials by specialty (orthopedic, emergency, elective surgery, etc).

An intrinsic problem in studies of VTE is the heterogeneous risk profile of individual participants. The lack of risk stratification in studies of VTE was recently highlighted in an extensive systematic review and meta-analysis of VTE rates after endovenous interventions for varicose veins, and it was recommended that risk stratification tools, such as the Caprini score, be adopted in future studies.^47,48^ In the context of this study, it may be that the participants with the highest risk of VTE stood to gain the most from combined GCS and pharmacological thromboprophylaxis, but unfortunately due to lack of standardized risk stratification, the role of GCS in this context remains uncertain. Although not answered here, a rolling Cochrane review of combined IPCD and pharmacological thromboprophylaxis has consistently demonstrated the benefits of the combined measures in patients with high risk and it is suggested that IPCD is used preferentially in these circumstances.^49^

GCS incurs a significant cost to health services, causes discomfort for patients, and in combination with pharmacological prophylaxis, does not significantly affect the rate of VTE nor VTE-related mortality as compared with pharmacological prophylaxis alone. Clinical guidelines, which recommend the use of GCS alongside pharmacological thromboprophylaxis for surgical inpatients, are not supported by the most recent literature.

## Supplementary Material
