# Supplementary material for: An Updated Systematic Review and Meta-analysis of the Impact of Graduated Compression Stockings in Addition to Pharmacological Thromboprophylaxis for Prevention of Venous Thromboembolism in Surgical Inpatients
Source: Ann Surg. 2023 Sep 27;279(1):29–36. doi: 10.1097/SLA.0000000000006096 (PMC10727201; doi:10.1097/SLA.0000000000006096)
Supplement: Supplementary file 1 [file sla-279-029-s001.docx]

| **Author**  **SUPPLEMENTAL TABLE 1**  **Table of baseline characteristics of the included studies** | **Year** | **Type of Surgery** | **Included Arm(s)** | **Thromboprophylaxis** | **Pharmacologic thromboprophylaxis** | **Duration of mechanical thromboprophylaxis** | **Duration of drug thromboprophylaxis, mean** | **Method of VTE diagnosis** | **Last Follow-up / End-point** | **Elective vs Emergency** |
| --- | --- | --- | --- | --- | --- | --- | --- | --- | --- | --- |
| Verhamme et al.^1^ | 2021 | Orthopaedic (knee replacement) | Single arm | Pharmacologic thromboprophylaxis alone | 40 mg enoxaparin OD | N/A | 8-12 days | US; venography | 30 days | Elective |
| Shalhoub et al. ^2^ | 2020 | Elective surgical procedures | Comparative, two arms included | GCS in conjunction with pharmacologic thromboprophylaxis vs. pharmacologic thromboprophylaxis alone | LMWH preparation varied by centre but accorded with manufacturer instructions | Until discharge | Until discharge | Duplex US; CTPA | 90 days | Elective |
| Guntupalli et al. ^3^ | 2020 | Gynecologic/ pelvic surgery | Single arm | Pharmacologic thromboprophylaxis alone | Initially 5000 units heparin calcium tds on day of surgery, then Enoxaparin 40mg OD | N/A | 28 days | US; CTPA | 90 days | Elective |
| Samama et al.^4^ | 2020 | Orthopaedic (non-major) | Single arm | Pharmacologic thromboprophylaxis alone | 4000 IU enoxaparin OD | N/A | 14 days - >2 months. Mean duration 28 days ±14 days (SD) | US; CTPA; V/Q scan; pulmonary angiography | Median follow up 33 days, interquartile range 31-34 days | Elective |
| Weitz et al.^5^ | 2020 | Orthopaedic (knee replacement) | Single arm | Pharmacologic thromboprophylaxis alone | Enoxaparin 40mg OD | N/A | 10-13 days | Bilateral venography | 10-13 days | Elective |
| Yu et al. ^6^ | 2020 | Gynaecologic/ pelvic surgery | Single arm | GCS in conjunction with pharmacologic thromboprophylaxis. | Enoxaparin 4250 IU OD | N/A | 28 days | US | 90 days | Elective |
| Huo et al.^7^ | 2019 | Orthopaedic (hip replacement) | Single arm | Pharmacologic thromboprophylaxis alone | LMWH-calcium 4100 units OD | N/A | 35 days | US | 35 days | Elective |
| Jiang et al.^8^ | 2019 | Orthopaedic (knee replacement) | Single arm | Pharmacologic thromboprophylaxis alone | LMWH 4000 IU OD | N/A | 35 days | US | 35 days | Elective |
| Sang et al.^9^ | 2018 | Gynecologic pelvic surgery | Single arm | GCS in conjunction with pharmacologic thromboprophylaxis | 5000 IU Dalteparin OD | Until ambulatory | 5 days | Duplex US | 30 days | Elective |
| Wang et al.^10^ | 2018 | Hepatobiliary (Primary liver cancer resection) | Single arm | Pharmacologic thromboprophylaxis alone | 5000 units Unipul OD | N/A | 2 - 7 days | Duplex US, CTPA | 10 days | Elective |
| Selby et al. ^11^ | 2015 | Orthopedic (fractures of tibia, fibula or ankle) | Single arm | Pharmacologic thromboprophylaxis alone | 5000 units dalteparin OD | N/A | 14 days | Duplex US, CTPA, V/Q scan | 3 months | Emergency |
| Du et al. ^12^ | 2015 | Orthopaedic (traumatic and elective cancer) | Single arm | Pharmacologic thromboprophylaxis alone | 40 mg parnaparin OD | N/A | 14 days | Duplex US, CTPA, | 28 days | Both |
| Kakkar et al.^13^ | 2014 | Major abdominal or pelvic | Single arm | Pharmacologic thromboprophylaxis alone | 40 mg enoxaparin OD | N/A | 7 - 10 days | Bilateral venography | 35 - 42 days | Both |
| Lassen at al. ^14^ | 2012 | Orthopedic (hip replacement) | Single arm | Pharmacologic thromboprophylaxis alone | 40 mg enoxaparin OD | N/A | 7-10 days | Bilateral venography; compression US; V/Q scan; CTPA | 42 days | Elective |
| Lassen at al.^14^ | 2012 | Orthopedic (hip fracture surgery) | Single arm | Pharmacologic thromboprophylaxis alone | 40 mg enoxaparin OD | N/A | 7-10 days | Bilateral venography; compression US; V/Q scan; CTPA | 42 days | Emergency |
| Lassen at al.^14^ | 2012 | Orthopedic (knee replacement) | Single arm | Pharmacologic thromboprophylaxis alone | 30 mg enoxaparin OD | N/A | 7-10 days | Bilateral venography; compression US; V/Q scan; CTPA | 42 days | Elective |
| Gomes et al.^15^ | 2011 | Major abdominal surgery | Single arm | Pharmacologic thromboprophylaxis alone | 40 mg enoxaparin OD | N/A | 7-10 days | Compression US; CT; pulmonary scintigraphy | 60 days | Both |
| Lassen at al.^16^ | 2010 | Orthopedic (knee replacement) | Single arm | Pharmacologic thromboprophylaxis alone | 40 mg enoxaparin OD | N/A | 12 days | Bilateral venography; compression US; V/Q scan; CTPA | 60 days | Elective |
| Colwell et al.^17^ | 2010 | Orthopedic (hip replacement) | Single arm | Pharmacologic thromboprophylaxis alone | 30 mg enoxaparin BD until discharge, then 40 mg OD | N/A | 10 days | US; CTPA | 10-12 days | Elective |
| Weitz et al.^18^ | 2010 | Orthopedic (knee replacement) | Single arm | Pharmacologic thromboprophylaxis alone | 30 mg enoxaparin BD | N/A | 10-14 days | Bilateral venography; V/Q scan; CTPA | 10 - 14 days (at last dose) | Elective |
| Raskob et al.^19^ | 2010 | Orthopedic (hip replacement) | Single arm | Pharmacologic thromboprophylaxis alone | Dalteparin: initial dose, 2500 IU; subsequent doses, 5000 IU OD | N/A | 7-10 days | Bilateral venography | 60 days (after last dose) | Elective |
| Turpie et al. ^20^ | 2009 | Orthopedic (knee arthroplasty) | Single arm | Pharmacologic thromboprophylaxis alone | 30 mg enoxaparin BD | N/A | 11-15 days | Bilateral venography; US; V/Q scan; CTPA; spiral CT | 35 days (post last dose) | Elective |
| Lassen at al. ^21^ | 2009 | Orthopedic (knee replacement) | Single arm | Pharmacologic thromboprophylaxis alone | 40 mg enoxaparin OD | N/A | 5-10 days | Bilateral venography | 30 days | Elective |
| Goel et al. ^22^ | 2009 | Orthopedic (tibial & ankle fractures) | Single arm | Pharmacologic thromboprophylaxis alone | Dalteparin: 2500 IU preoperatively; 5000 IU postoperatively OD | N/A | 14 days | Bilateral venography; US | 90 days | Emergency |
| Kakkar et al.^23^ | 2008 | Orthopedic (hip replacement) | Single arm | Pharmacologic thromboprophylaxis alone | 40 mg enoxaparin OD | N/A | 12.4 days | Bilateral venography; US; V/Q scan; CTPA; spiral CT | 32 - 40 days | Elective |
| Cohen et al.^24^ | 2007 | Orthopaedic (Hip) | Comparative, two arms included | GCS in conjunction with pharmacologic thromboprophylaxis vs. pharmacologic thromboprophylaxis alone | 2.5 mg fondaparinux OD | 42 days | Fixed, 7 days | Duplex US, Venography | 42 days | Both |
| Lassen at al. ^25^ | 2007 | Orthopedic (knee replacement) | Single arm | Pharmacologic thromboprophylaxis alone | 30 mg enoxaparin BD | N/A | 12 days | Venography; V/Q scan; spiral CT; US | 30 days (after last dose) | Elective |
| Rasmussen et al.^26^ | 2006 | Major abdominal surgery | Single arm | GCS in conjunction with pharmacologic thromboprophylaxis. | 5000 IU dalteparin OD | 7 days | 7 days | Venography; V/Q scan; spiral CT | 28 days | Both |
| Eriksson et al.^27^ | 2006 | Orthopedic (hip replacement) | Single arm | Pharmacologic thromboprophylaxis alone | 40 mg enoxaparin OD | N/A | 5 - 9 days | Venography; V/Q scan; spiral CT | 60 days (post last dose) | Elective |
| Howard et al. ^28^ | 2004 | Breast, oncology, ENT, GI, neurosurgery, orthopedic, urology, varicose vein | Single arm | GCS in conjunction with pharmacologic thromboprophylaxis. | 20 mg enoxaparin OD | 5-7 days | Until discharge, 5-7 days | Duplex US | 5- 7 days | Both |
| Pitto et al. ^29^ | 2004 | Orthopedic (hip replacement) | Single arm | GCS in conjunction with pharmacologic thromboprophylaxis. | 1900 IU-5700 IU nadroparin OD | Until discharge, 13 days | Until discharge, 13 days | Duplex US | 45 days | Elective |

**SUPPLEMENTAL TABLE 1 LEGEND**

Supplemental Table 1 – the baseline characteristics of the included studies. Abbreviations list: twice a day (BD); computed tomography (CT); computed tomography pulmonary angiogram (CTPA); graduated compression stockings (GCS); international units (IU); low molecular weight heparin (LMWH); not applicable (N/A); once a day (OD); standard deviation (SD); ultrasound (US); ventilation-perfusion (V/Q); venous thromboembolism (VTE)

**REFERENCES**

1. Verhamme P, Yi BA, Segers A, et al. Abelacimab for Prevention of Venous Thromboembolism. *N Engl J Med* 2021; 385(7):609-617.

2. Shalhoub J, Lawton R, Hudson J, et al. Graduated compression stockings as adjuvant to pharmaco-thromboprophylaxis in elective surgical patients (GAPS study): Randomised controlled trial. *The BMJ* 2020; 369 (no pagination).

3. Guntupalli SR, Brennecke A, Behbakht K, et al. Safety and Efficacy of Apixaban vs Enoxaparin for Preventing Postoperative Venous Thromboembolism in Women Undergoing Surgery for Gynecologic Malignant Neoplasm: A Randomized Clinical Trial. *JAMA Netw Open* 2020; 3(6):e207410.

4. Samama CM, Laporte S, Rosencher N, et al. Rivaroxaban or Enoxaparin in Nonmajor Orthopedic Surgery. *N Engl J Med* 2020; 382(20):1916-1925.

5. Weitz JI, Bauersachs R, Becker B, et al. Effect of Osocimab in Preventing Venous Thromboembolism Among Patients Undergoing Knee Arthroplasty: The FOXTROT Randomized Clinical Trial. *JAMA* 2020; 323(2):130-139.

6. Yu R, Nansubuga F, Yang J, et al. Efficiency and safety evaluation of prophylaxes for venous thrombosis after gynecological surgery. *Medicine (Baltimore)* 2020; 99(25):e20928.

7. Huo JL, M; Liu, B; Liu, J; Ma, W; Wu, T; Li, H; Han, Y. A comparison of indobufen and low-molecular-weight heparin in the prevention of deep vein thrombosis in patients after total hip arthroplasty: a prospective randomized controlled study. *International journal of clinical and experimental medicine* 2019; 12(3):2720-2728.

8. Jiang H, Meng J, Guo T, et al. Comparison of Apixaban and Low Molecular Weight Heparin in Preventing Deep Venous Thrombosis after Total Knee Arthroplasty in Older Adults. *Yonsei Med J* 2019; 60(7):626-632.

9. Sang CQ, Zhao N, Zhang J, et al. Different combination strategies for prophylaxis of venous thromboembolism in patients: A prospective multicenter randomized controlled study. *Sci Rep* 2018; 8(1):8277.

10. Wang YH, Qiu H, He XL, et al. Prevention of venous thromboembolism after resection of primary liver cancer with low molecular weight heparin and its association with P-selectin, lysosomal granule glycoprotein, platelet activating factor and plasma D-dimer. *Eur Rev Med Pharmacol Sci* 2018; 22(14):4657-4662.

11. Selby R, Geerts WH, Kreder HJ, et al. A double-blind, randomized controlled trial of the prevention of clinically important venous thromboembolism after isolated lower leg fractures. *J Orthop Trauma* 2015; 29(5):224-30.

12. Du W, Zhao C, Wang J, et al. Comparison of rivaroxaban and parnaparin for preventing venous thromboembolism after lumbar spine surgery. *J Orthop Surg Res* 2015; 10:78.

13. Kakkar AK, Agnelli G, Fisher W, et al. Preoperative enoxaparin versus postoperative semuloparin thromboprophylaxis in major abdominal surgery: a randomized controlled trial. *Ann Surg* 2014; 259(6):1073-9.

14. Lassen MR, Fisher W, Mouret P, et al. Semuloparin for prevention of venous thromboembolism after major orthopedic surgery: results from three randomized clinical trials, SAVE-HIP1, SAVE-HIP2 and SAVE-KNEE. *J Thromb Haemost* 2012; 10(5):822-32.

15. Gomes M, Ramacciotti E, Henriques AC, et al. Generic versus branded enoxaparin in the prevention of venous thromboembolism following major abdominal surgery: report of an exploratory clinical trial. *Clin Appl Thromb Hemost* 2011; 17(6):633-9.

16. Lassen MR, Raskob GE, Gallus A, et al. Apixaban versus enoxaparin for thromboprophylaxis after knee replacement (ADVANCE-2): a randomised double-blind trial. *Lancet* 2010; 375(9717):807-15.

17. Colwell CW, Jr., Froimson MI, Mont MA, et al. Thrombosis prevention after total hip arthroplasty: a prospective, randomized trial comparing a mobile compression device with low-molecular-weight heparin. *J Bone Joint Surg Am* 2010; 92(3):527-35.

18. Weitz JI, Cao C, Eriksson BI, et al. A dose-finding study with TAK-442, an oral factor Xa inhibitor, in patients undergoing elective total knee replacement surgery. *Thromb Haemost* 2010; 104(6):1150-7.

19. Raskob G, Cohen AT, Eriksson BI, et al. Oral direct factor Xa inhibition with edoxaban for thromboprophylaxis after elective total hip replacement. A randomised double-blind dose-response study. *Thromb Haemost* 2010; 104(3):642-9.

20. Turpie AG, Lassen MR, Davidson BL, et al. Rivaroxaban versus enoxaparin for thromboprophylaxis after total knee arthroplasty (RECORD4): a randomised trial. *Lancet* 2009; 373(9676):1673-80.

21. Lassen MR, Dahl OE, Mismetti P, et al. AVE5026, a new hemisynthetic ultra-low-molecular-weight heparin for the prevention of venous thromboembolism in patients after total knee replacement surgery--TREK: a dose-ranging study. *J Thromb Haemost* 2009; 7(4):566-72.

22. Goel DP, Buckley R, deVries G, et al. Prophylaxis of deep-vein thrombosis in fractures below the knee: a prospective randomised controlled trial. *J Bone Joint Surg Br* 2009; 91(3):388-94.

23. Kakkar AK, Brenner B, Dahl OE, et al. Extended duration rivaroxaban versus short-term enoxaparin for the prevention of venous thromboembolism after total hip arthroplasty: a double-blind, randomised controlled trial. *Lancet* 2008; 372(9632):31-9.

24. Cohen AT, Skinner JA, Warwick D, et al. The use of graduated compression stockings in association with fondaparinux in surgery of the hip. A multicentre, multinational, randomised, open-label, parallel-group comparative study. *J Bone Joint Surg Br* 2007; 89(7):887-92.

25. Lassen MR, Davidson BL, Gallus A, et al. The efficacy and safety of apixaban, an oral, direct factor Xa inhibitor, as thromboprophylaxis in patients following total knee replacement. *J Thromb Haemost* 2007; 5(12):2368-75.

26. Rasmussen MS, Jorgensen LN, Wille-Jorgensen P, et al. Prolonged prophylaxis with dalteparin to prevent late thromboembolic complications in patients undergoing major abdominal surgery: a multicenter randomized open-label study. *J Thromb Haemost* 2006; 4(11):2384-90.

27. Eriksson BI, Borris LC, Dahl OE, et al. A once-daily, oral, direct Factor Xa inhibitor, rivaroxaban (BAY 59-7939), for thromboprophylaxis after total hip replacement. *Circulation* 2006; 114(22):2374-81.

28. Howard A, Zaccagnini D, Ellis M, et al. Randomized clinical trial of low molecular weight heparin with thigh-length or knee-length antiembolism stockings for patients undergoing surgery. *Br J Surg* 2004; 91(7):842-7.

29. Pitto RP, Hamer H, Heiss-Dunlop W, et al. Mechanical prophylaxis of deep-vein thrombosis after total hip replacement a randomised clinical trial. *J Bone Joint Surg Br* 2004; 86(5):639-42.

**SUPPLEMENTAL TABLE 2**

| **Author** | **Random sequence generation** | **Allocation concealment** | **Blinding (participants and personnel)** | **Blinding (outcome assessment)** | **Incomplete outcome data** | **Selective reporting** | **Other sources of bias** | **Overall** | **Notes** |
| --- | --- | --- | --- | --- | --- | --- | --- | --- | --- |
| Cohen et al. | Unclear | Low | High | Low | Unclear | Low | Low | Unclear | Single-blind |
| Sang et al. | Low | Unclear | High | High | High | Unclear | Low | High | Unblinded, immediate symptomatic imaging, 6 of 32 patients with DVT did not undergo CTPA |
| Howard et al. | Unclear | Unclear | High | Unclear | High | Unclear | Low | High | Unblinded, average trial completion of 78.2% |
| Rasmussen et al. | Unclear | Low | High | Low | Low | Unclear | Low | High | Single-blind, reasons for exclusions stated |
| Pitto et al. | Unclear | Low | High | Low | Low | unclear | Low | High | Single-blind, exclusion explained adequately |
| Wang et al. | Low | Unclear | High | High | Low | Unclear | Low | High | Unblinded, symptomatic imaging |
| Selby et al. | Low | Low | low | Low | Low | Unclear | Low | Unclear | Double-blinded, exclusion explained adequately |
| Kakkar et al. | low | Unclear | low | Unclear | Low | Unclear | Low | Unclear | Double-blinded, exclusion explained adequately |
| Du et al. | Low | Unclear | High | High | High | Unclear | Low | High | 120 patients lost to follow-up with no explanation |
| Lassen at al. | Low | Unclear | low | Low | Low | Unclear | Low | Unclear | Double-blinded, exclusion explained adequately |
| Lassen at al. | Low | Unclear | low | Low | Low | Unclear | Low | Unclear | Double-blinded, exclusion explained adequately |
| Lassen at al. | Low | Unclear | low | Low | Low | Unclear | Low | Unclear | Double-blinded, exclusion explained adequately |
| Gomes et al. | Low | high | High | High | High | Unclear | Low | High | 30 unexplained post-randomisation exclusions |
| Lassen at al. | Low | Low | low | Low | Low | Unclear | Low | Unclear | Double-blinded, exclusion explained adequately |
| Colwell et al. | Low | High | High | Low | Low | Unclear | Low | High | Unblinded |
| Turpie et al. | Low | Low | Low | Low | Low | Low | Low | Low | Double-blinded, exclusion explained adequately |
| Lassen at al. | Low | Unclear | Low | Low | Unclear | Unclear | Low | High | Double blinded but only 67.2% of randomised patients were included in the primary efficacy analysis. Women were excluded if not using effective contraception |
| Kakkar et al. | Low | Low | Low | Low | Low | Low | Low | Low | Double-blinded, exclusion explained adequately |
| Eriksson et al. | Unclear | Unclear | Low | Low | High | Unclear | Low | High | Double-blinded, one third of participants excluded from efficacy analysis |
| Weitz et al. | Low | Low | Low | Low | Low | Unclear | Low | Unclear | Single blinded, knowledge of intervention by patients unlikely to affect outcome |
| Raskob et al. | Low | Low | Low | Low | Low | Low | Low | Low | Double-blinded, exclusion explained adequately |
| Goel et al. | Low | Unclear | Low | Low | Low | Unclear | Low | Unclear | Double-blinded, exclusion explained adequately, protocol mentioned but details not given |
| Lassen at al. | Low | Low | Low | Low | Unclear | High | Low | High | Double blinded but only 67.2% of randomised patients were included in the primary efficacy analysis. Women were excluded if not using effective contraceptionWomen of child-bearing age exlcuded, difficult to generalise. No mention of a protocol. |
| Huo et al. | Unclear | Unclear | High | High | Low | Low | Low | High | Unblinded, poor study design |
| Jiang et al. | Unclear | High | High | High | Unclear | Unclear | Low | High | Unblinded, poor study design |
| Guntupalli et al | Low | Low | High | Low | Unclear | Low | Low | High | Open-label, unclear in which group the attrition occurred and whether it was balanced between the groups |
| Samama et al. | Low | Low | Low | Low | Low | Low | Low | Low | Double-blinded, exclusion explained adequately |
| Shahloub et al. | Low | Low | Low | Low | Low | Low | Low | Low | Open label but the wearing of stockings unlikely to have affected outcome of VTE. 95.7% received GCS |
| Weitz et al. | Low | Low | Low | Low | Low | Low | Low | Low | Single blinded but knowledge of treatment unlikely to affect outcome of VTE |
| Yu et al. | Low | High | Low | Low | Low | Low | Low | High | Single blinded, knowledge of intervention by patients unlikely to affect outcome. No allocation concealment described |
| Verhamme et al. | Unclear | Unclear | Low | Low | Low | Low | Low | Unclear | Randomisation stratified by centre, unclear whether this could have affected outcomes. Single blinded, knowledge of intervention by patients unlikely to affect outcome |

**SUPPLEMENTAL TABLE 2 LEGEND**

Supplemental Table 2 – the risk of bias assessment for randomised trials included in the systematic review and meta-analysis.

**SUPPLEMENTAL DATA FILE FOR JOURNAL ONLY – PRISMA 2020 CHECKLIST**


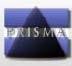
 **PRISMA 2020 Checklist**

| **Section and Topic** | **Item #** | **Checklist item** | **Location where item is reported** |
| --- | --- | --- | --- |
| **TITLE** | | |  |
| Title | 1 | Identify the report as a systematic review. | Title, page 1 |
| **ABSTRACT** | | |  |
| Abstract | 2 | See the PRISMA 2020 for Abstracts checklist. | Abstract, pages 3-4 |
| **INTRODUCTION** | | |  |
| Rationale | 3 | Describe the rationale for the review in the context of existing knowledge. | Introduction, page 7 |
| Objectives | 4 | Provide an explicit statement of the objective(s) or question(s) the review addresses. | Abstract, page 7; introduction page 7 |
| **METHODS** | | |  |
| Eligibility criteria | 5 | Specify the inclusion and exclusion criteria for the review and how studies were grouped for the syntheses. | Methods, pages 8-9 |
| Information sources | 6 | Specify all databases, registers, websites, organisations, reference lists and other sources searched or consulted to identify studies. Specify the date when each source was last searched or consulted. | Methods, page 8 |
| Search strategy | 7 | Present the full search strategies for all databases, registers and websites, including any filters and limits used. | Page 9, refers to search terms used for previous review |
| Selection process | 8 | Specify the methods used to decide whether a study met the inclusion criteria of the review, including how many reviewers screened each record and each report retrieved, whether they worked independently, and if applicable, details of automation tools used in the process. | Page 8 |
| Data collection process | 9 | Specify the methods used to collect data from reports, including how many reviewers collected data from each report, whether they worked independently, any processes for obtaining or confirming data from study investigators, and if applicable, details of automation tools used in the process. | Page 9 |
| Data items | 10a | List and define all outcomes for which data were sought. Specify whether all results that were compatible with each outcome domain in each study were sought (e.g. for all measures, time points, analyses), and if not, the methods used to decide which results to collect. | Page 9 |
|  | 10b | List and define all other variables for which data were sought (e.g. participant and intervention characteristics, funding sources). Describe any assumptions made about any missing or unclear information. | Page 9 |
| Study risk of bias assessment | 11 | Specify the methods used to assess risk of bias in the included studies, including details of the tool(s) used, how many reviewers assessed each study and whether they worked independently, and if applicable, details of automation tools used in the process. | Page 9 |
| Effect measures | 12 | Specify for each outcome the effect measure(s) (e.g. risk ratio, mean difference) used in the synthesis or presentation of results. | Page 9 |
| Synthesis methods | 13a | Describe the processes used to decide which studies were eligible for each synthesis (e.g. tabulating the study intervention characteristics and comparing against the planned groups for each synthesis (item #5)). | Page 9 |
|  | 13b | Describe any methods required to prepare the data for presentation or synthesis, such as handling of missing summary statistics, or data conversions. | Page 9 |
|  | 13c | Describe any methods used to tabulate or visually display results of individual studies and syntheses. | Page 9 |
|  | 13d | Describe any methods used to synthesize results and provide a rationale for the choice(s). If meta-analysis was performed, describe the model(s), method(s) to identify the presence and extent of statistical heterogeneity, and software package(s) used. | Page 9 |
|  | 13e | Describe any methods used to explore possible causes of heterogeneity among study results (e.g. subgroup analysis, meta-regression). | Page 9 |
|  | 13f | Describe any sensitivity analyses conducted to assess robustness of the synthesized results. | N/A |
| Reporting bias assessment | 14 | Describe any methods used to assess risk of bias due to missing results in a synthesis (arising from reporting biases). | Page 9 |
| Certainty assessment | 15 | Describe any methods used to assess certainty (or confidence) in the body of evidence for an outcome. | Page 9 |


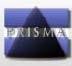
 **PRISMA 2020 Checklist**

| **Section and Topic** | **Item #** | **Checklist item** | **Location where item is reported** |
| --- | --- | --- | --- |
| **RESULTS** | |  |  |
| Study selection | 16a | Describe the results of the search and selection process, from the number of records identified in the search to the number of studies included in the review, ideally using a flow diagram. | Page 10 |
|  | 16b | Cite studies that might appear to meet the inclusion criteria, but which were excluded, and explain why they were excluded. | Page 10 |
| Study characteristics | 17 | Cite each included study and present its characteristics. | Page 10 |
| Risk of bias in studies | 18 | Present assessments of risk of bias for each included study. | Page 13 |
| Results of individual studies | 19 | For all outcomes, present, for each study: (a) summary statistics for each group (where appropriate) and (b) an effect estimate and its precision (e.g. confidence/credible interval), ideally using structured tables or plots. | Pages 11-13 and figures 2-5 |
| Results of syntheses | 20a | For each synthesis, briefly summarise the characteristics and risk of bias among contributing studies. | Pages 11-13 |
|  | 20b | Present results of all statistical syntheses conducted. If meta-analysis was done, present for each the summary estimate and its precision (e.g.  confidence/credible interval) and measures of statistical heterogeneity. If comparing groups, describe the direction of the effect. | Pages 11-13 |
|  | 20c | Present results of all investigations of possible causes of heterogeneity among study results. | Pages 11-13 |
|  | 20d | Present results of all sensitivity analyses conducted to assess the robustness of the synthesized results. | N/A |
| Reporting biases | 21 | Present assessments of risk of bias due to missing results (arising from reporting biases) for each synthesis assessed. | Page 13 |
| Certainty of evidence | 22 | Present assessments of certainty (or confidence) in the body of evidence for each outcome assessed. | Page 13 |
| **DISCUSSION** | |  |  |
| Discussion | 23a | Provide a general interpretation of the results in the context of other evidence. | Page 14 |
|  | 23b | Discuss any limitations of the evidence included in the review. | Page 17 |
|  | 23c | Discuss any limitations of the review processes used. | Page 17 |
|  | 23d | Discuss implications of the results for practice, policy, and future research. | Pages 15-17 |
| **OTHER INFORMATION** | |  |  |
| Registration and protocol | 24a | Provide registration information for the review, including register name and registration number, or state that the review was not registered. | Page 8 |
|  | 24b | Indicate where the review protocol can be accessed, or state that a protocol was not prepared. | Page 8 |
|  | 24c | Describe and explain any amendments to information provided at registration or in the protocol. | Page 8 |
| Support | 25 | Describe sources of financial or non-financial support for the review, and the role of the funders or sponsors in the review. | Title page |
| Competing  interests | 26 | Declare any competing interests of review authors. | Title page |
| Availability of data, code and other materials | 27 | Report which of the following are publicly available and where they can be found: template data collection forms; data extracted from included studies; data used for all analyses; analytic code; any other materials used in the review. | Page 10 |

*From:*  Page MJ, McKenzie JE, Bossuyt PM, Boutron I, Hoffmann TC, Mulrow CD, et al. The PRISMA 2020 statement: an updated guideline for reporting systematic reviews. BMJ 2021;372:n71. doi:

10.1136/bmj.n71

For more information, visit:<http://www.prisma-statement.org/>

**SUPPLEMENTAL DATA FILE LEGEND**

The PRISMA 2020 checklist indicating how the review was conducted and the location of information within the manuscript.
